# NDP-MSH binding melanocortin-1 receptor ameliorates neuroinflammation and BBB disruption through CREB/Nr4a1/NF-κB pathway after intracerebral hemorrhage in mice

**DOI:** 10.1186/s12974-019-1591-4

**Published:** 2019-10-28

**Authors:** Xuan Wu, Siming Fu, Yun Liu, Hansheng Luo, Feng Li, Yiying Wang, Meng Gao, Yuan Cheng, Zongyi Xie

**Affiliations:** 1 Department of Neurosurgery, The Second Affiliated Hospital, Chongqing Medical University, 76th Linjiang Road, Yuzhong District, Chongqing, 400010 China; 2 Department of Human Anatomy, Chongqing Medical University, Chongqing, 400016 China

**Keywords:** Intracerebral hemorrhage, Neuroinflammation, Blood-brain barrier, NDP-MSH, Mc1r, Nr4a1

## Abstract

**Background:**

Neuroinflammation and blood-brain barrier (BBB) disruption are two vital mechanisms of secondary brain injury following intracerebral hemorrhage (ICH). Recently, melanocortin-1 receptor (Mc1r) activation by Nle4-D-Phe7-α-MSH (NDP-MSH) was shown to play a neuroprotective role in an experimental autoimmune encephalomyelitis (EAE) mouse model. This study aimed to investigate whether NDP-MSH could alleviate neuroinflammation and BBB disruption after experimental ICH, as well as the potential mechanisms of its neuroprotective roles.

**Methods:**

Two hundred and eighteen male C57BL/6 mice were subjected to autologous blood-injection ICH model. NDP-MSH, an agonist of Mc1r, was administered intraperitoneally injected at 1 h after ICH insult. To further explore the related protective mechanisms, Mc1r small interfering RNA (Mc1r siRNA) and nuclear receptor subfamily 4 group A member 1 (Nr4a1) siRNA were administered via intracerebroventricular (i.c.v) injection before ICH induction. Neurological test, BBB permeability, brain water content, immunofluorescence staining, and Western blot analysis were implemented.

**Results:**

The Expression of Mc1r was significantly increased after ICH. Mc1r was mainly expressed in microglia, astrocytes, and endothelial cells following ICH. Treatment with NDP-MSH remarkably improved neurological function and reduced BBB disruption, brain water content, and the number of microglia in the peri-hematoma tissue after ICH. Meanwhile, the administration of NDP-MSH significantly reduced the expression of p-NF-κB p65, IL-1β, TNF-α, and MMP-9 and increased the expression of p-CREB, Nr4a1, ZO-1, occludin, and Lama5. Inversely, the knockdown of Mc1r or Nr4a1 abolished the neuroprotective effects of NDP-MSH.

**Conclusions:**

Taken together, NDP-MSH binding Mc1r attenuated neuroinflammation and BBB disruption and improved neurological deficits, at least in part through CREB/Nr4a1/NF-κB pathway after ICH.

## Background

Intracerebral hemorrhage (ICH) is a severe cerebral vascular disease with high morbidity and mortality, and its incidence is increasing annually [1]. Mounting evidence has demonstrated that neuroinflammation and blood-brain barrier (BBB) disruption are two critical mechanisms of ICH-induced brain injury, which are closely associated with poor prognosis [2]. Therefore, a therapeutic strategy targeting neuroinflammation and BBB disruption would be beneficial for attenuating brain injury following ICH.

The neuropeptide α-melanocyte-stimulating hormone (α-MSH) is a member of the melanocortin family, a group of peptides derived from pro-opiomelanocortin (POMC) [3]. α-MSH exerts well-established roles in the regulation of skin pigmentation and energy homeostasis, as well as inflammatory reaction [4, 5]. The biological function of α-MSH is mediated by five melanocortin receptors (termed Mc1r to Mc5r) [6]. Melanocortin-1 receptor (Mc1r), a G protein-coupled receptor, is best known as a mediator of the synthesis of melanin pigments, and it is also implicated in inflammation which is regulated by NF-κB signaling pathway [7–9]. α-MSH is released from cells in the central nervous system; however, the chemical property of α-MSH is unstable, transformed into the protease-stable Nle4-D-Phe7-α-MSH (NDP-MSH), which has a specific higher affinity to Mc1r [8, 10, 11]. Treatment with NDP-MSH was proven to reduce inflammation and vasospasm after subarachnoid hemorrhage [12]. Likewise, the administration of NDP-MSH ameliorated blood-brain barrier (BBB) disruption by activating Mc1r in a model of experimental autoimmune encephalomyelitis (EAE) [13]. Despite the well-recognized roles of NDP-MSH and Mc1r on inflammation, the effects of NDP-MSH and Mc1r on neuroinflammation and BBB integrity after ICH have not been elucidated.

Nuclear receptor subfamily 4 group A member 1 (Nr4a1), a member of Nur nuclear receptor family of transcriptional factors, is involved in neuroinflammation as a regulator of microglia activation in EAE in mice [14]. A previous study indicated that Nr4a1 was induced and functions immediately downstream of Mc1r signaling in melanocytic cells [15]. Furthermore, Mykicki et al. showed that NDP-MSH binding to Mc1r initiated the phosphorylation of cAMP response element-binding protein (CREB), and activated Nr4a1, subsequently exerted long-lasting neuroprotective roles in mice with EAE [13]. It was reported that Nr4a orphan receptors could regulate NF-κB signaling in microglial and myeloid cells [16, 17]. Moreover, mounting evidence revealed that Nr4a1 negatively modulated the transcriptional activity of NF-κB and inhibited inflammatory gene expression [18–21].

In the present study, we hypothesized that Mc1r activation by NDP-MSH could attenuate neuroinflammation and preserve BBB integrity after experimental ICH, and the protective mechanism is mediated through CREB/Nr4a1/NF-κB pathway.

## Methods

### Animals

All experimental protocols for this study were approved by the Animal Ethics Committee of Chongqing Medical University. The study complied with the National Institutes of Health guide for the care and use of Laboratory Animals and the ARRIVE (Animal Research: Reporting In Vivo Experiments) guidelines. A total of 218 C57BL/6 mice (male, weight about 25 g) were purchased from and bred at the Animal Center of Chongqing Medical University. All mice were housed in a light- and temperature-controlled room with free access to food and water.

### Experimental design

**Fig. 1:**
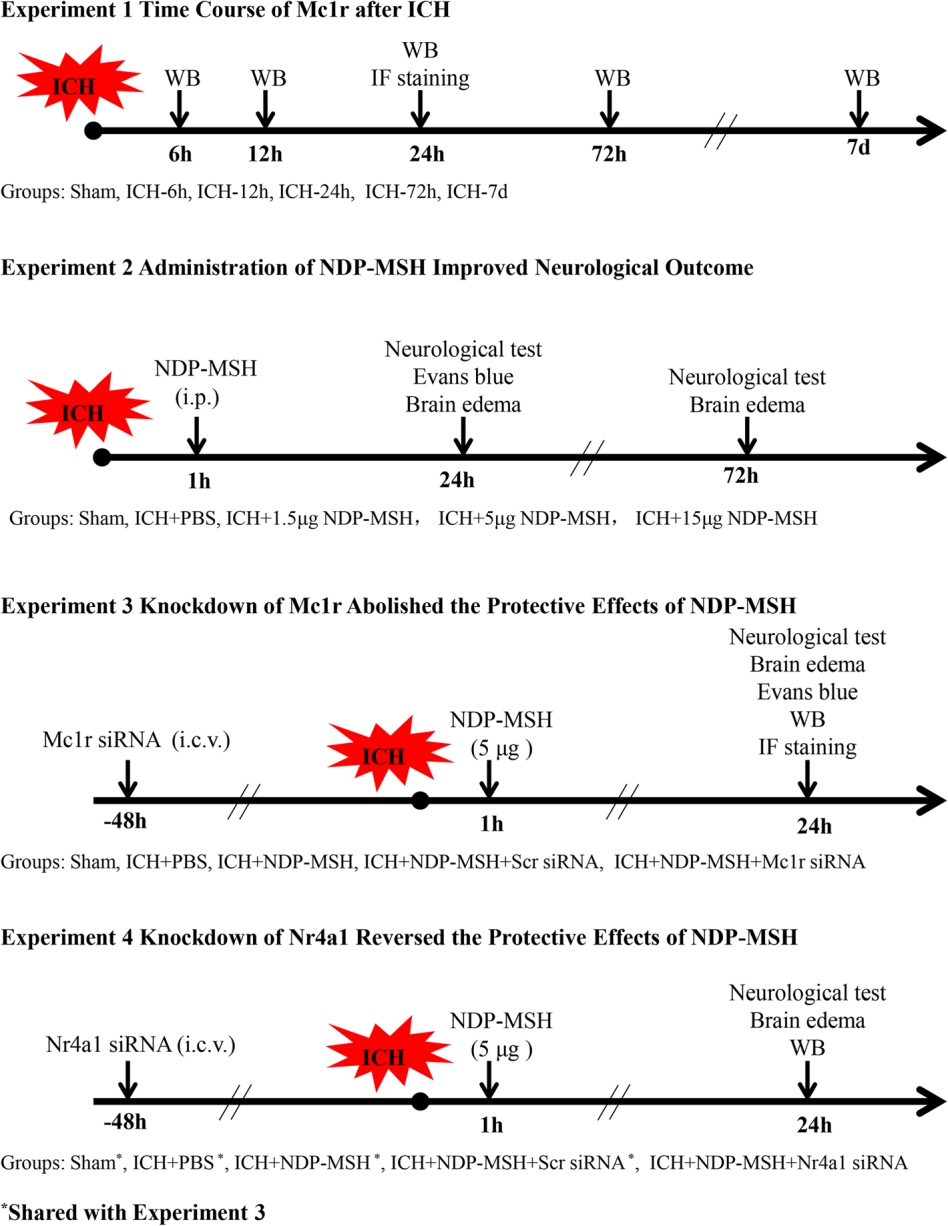
Experimental design and animal groups. ICH, intracerebral hemorrhage; Mc1r, melanocortin-1 receptor; Nr4a1, nuclear receptor subfamily 4 group A member 1; IF staining, immunofluorescence staining; WB, Western blot; Scr siRNA, scrambled siRNA

Four separate experiments were designed as follows (Fig. 1). A total of 218 mice were used (Additional file 1: Table S1).

#### Experiment 1

The time course of endogenous Mc1r in the peri-hematoma tissue was measured by Western blot. The cellular localization of Mc1r was detected by double immunofluorescence staining at 24 h after ICH.

#### Experiment 2

To evaluate the effects of NDP-MSH on neuroinflammation and BBB integrity, three doses of NDP-MSH (1.5, 5, 15 μg/mouse, Anaspec, USA) dissolved in phosphate-buffered saline (PBS) were administered intraperitoneally at 1 h after ICH insult. Mice were randomly divided into five groups: sham, ICH + vehicle (PBS), ICH + NDP-MSH (1.5 μg/mouse), ICH + NDP-MSH (5 μg/mouse), and ICH + NDP-MSH (15 μg/mouse). Neurological test and brain water content were examined at 24 and 72 h after ICH. Evans blue (EB) extravasation was evaluated at 24 h after ICH.

#### Experiment 3

To assess the effect of in vivo knockdown of Mc1r on neuroinflammation and BBB permeability, Mc1r small interfering RNA (siRNA) was administered by intracerebroventricular (i.c.v) injection at 48 h before ICH induction, and then followed with NDP-MSH (5 μg/mouse) treatment at 1 h after ICH. Neurological test, brain water content, EB extravasation, immunofluorescence staining, and Western blot were carried out at 24 h post-ICH. Mice were randomly divided into five groups: sham, ICH + vehicle (PBS), ICH + NDP-MSH (5 μg/mouse), ICH + NDP-MSH (5 μg/mouse) + scrambled siRNA (Scr siRNA), and ICH + NDP-MSH (5 μg/mouse) + Mc1r siRNA. In addition, to verify the knockdown efficiency of Mc1r siRNA, the expression of Mc1r in the right hemisphere was analyzed by Western blot. Mice were randomly assigned to four groups: Naive+Scr siRNA, Naive+Mc1r siRNA, ICH + Scr siRNA, and ICH + Mc1r siRNA.

#### Experiment 4

To investigate the underlying mechanisms of NDP-MSH-mediated neuroprotective effects, Nr4a1 siRNA was administered by i.c.v injection at 48 h before ICH induction, and then followed with NDP-MSH (5 μg/mouse) treatment at 1 h after ICH. Neurological test, brain water content, and Western blot were implemented at 24 h following ICH. Mice were randomly allotted into five groups: sham, ICH + vehicle, ICH + NDP-MSH (5 μg/mouse), ICH + NDP-MSH (5 μg/mouse) + Scr siRNA, and ICH + DNP-MSH (5 μg/mouse) + Nr4a1 siRNA. Moreover, to validate the knockdown efficiency of Nr4a1 siRNA, the expression of Nr4a1 was measured by Western blot. Mice were randomly assigned to four groups: Naive + Scr siRNA, Naive + Nr4a1 siRNA, ICH + Scr siRNA, and ICH + Nr4a1 siRNA.

### ICH mouse model induction

The ICH model was induced by autologous blood injection as previously described [22]. Briefly, the mice were anesthetized and fixed prone in a stereotaxic frame. Drill a small hole about 1 mm in diameter at 2 mm to the right of the bregma. Then 30 μl autologous arterial blood without anticoagulation was drawn from the central artery of the tail and delivered into the basal ganglion (stereotaxic coordinates: 0.2 mm anterior, 2.3 mm right lateral to the bregma, and 3.5 mm ventral to the skull). Firstly, 5 μl of blood was injected at 0.7 mm above the target position. Five minutes later, the remaining 25 μl blood was delivered at 3.5 mm depth. The needle was left for 10 min more after injection and withdrawn slowly at a rate of 1 mm/min. Bone wax was then applied to cover the drilled hole. The sham-operated animals were delivered an equal volume of sterile saline at the same position.

### Intracerebroventricular injection

Intracerebroventricular injection was performed as previously described [23]. Briefly, mice were anesthetized and placed in a stereotactic head frame in the prone position. A longitudinal incision was made along the midline and a burr hole was drilled to the right of the bregma (1.0 mm lateral of the bregma). Following the manufacturer’s instructions, Mc1r siRNA (Thermo Fisher Scientific, USA, MSS275666, GCG AUU CUG UAU GCC CAC AUG UUC A, UGA ACA UGU GGG CAU ACA GAA UCG C), Nr4a1 siRNA (Thermo Fisher Scientific, USA, MSS205160, GAA GAU GCC GGU GAC GUG CAA CAA U, AUU GUU GCA CGU CAC CGG CAU CUU C), or scramble siRNA was dissolved in sterile RNase-free water. Mc1r siRNA mixture or scramble siRNA (100 pmol/2 μl) was delivered into the ipsilateral ventricle at the depth of 2.5 mm. The needle was left for an additional 5 min after injection to avert possible leakage and was slowly withdrawn at a rate of 1 mm/min. The burr hole was sealed with bone wax, and the incision was closed with sutures. Mice were placed in an individual recovery cage.

### Neurobehavioral function test

Neurobehavioral functions were evaluated using the modified Garcia test and corner turn test at 24 or 72 h following ICH by a blinded investigator as previously described [24]. In the modified Garcia test, seven items including spontaneous activity, axial sensation, vibrissae touch, limb symmetry, lateral turning, forelimb walking, and climbing were tested. In the corner turn test, mice were allowed to approach a 30° corner. The mice exited the corner with either a right turn or left turn. Ten trials were performed, with at least a 30-s break between the trials. The percentage of a right turn to 10 trials was then calculated.

### BBB permeability

To evaluate BBB permeability, Evans blue (Aladdin, China) was injected intraperitoneally (100 μl of 4% solution in saline) as previously described with a slight modification [25]. After 3 h circulation, mice were transcardially perfused with cold phosphate-buffered saline (0.1 M, PBS, pH 7.4) under deep anesthesia. Afterwards, the brain was removed and divided into left and right hemispheres and stored at − 80 °C immediately. The right part of the brain was homogenized in 1100 μl PBS, sonicated, and centrifuged (12,000 g, 4 °C, 30 min). The supernatant was collected and added an equal amount of trichloroacetic acid (TCA) to incubate overnight by 4 °C. After centrifugation (12,000 g, 4 °C, 30 min), Evans blue stain was measured by spectrophotometer (Thermo Fisher Scientific, USA) at 610 nm.

### Brain water content

Brain water content was measured at 24 h and 72 h after ICH by an investigator blind to group information as previously described [26]. In short, mice were sacrificed under deep anesthesia. The brain was immediately removed and cut into 4 mm coronal slice. The brain slice was separated into five parts: ipsilateral and contralateral basal ganglia, ipsilateral and contralateral cortex, and cerebellum. The cerebellum was retained as an internal control. Each part was immediately weighed on an electronic analytical balance (FA2204B, Techcomp, USA) to determine the wet weight (WW) and then dried at 100 °C for 72 h to determine the dry weight (DW). Brain water content (percentage) was calculated as [(WW − DW)/WW] × 100%.

### Immunofluorescence staining

Double fluorescence staining was performed as described previously [27]. The mice were deeply anesthetized and were transcardially perfused with 20 ml ice-cold PBS followed by 20 ml of 4% paraformaldehyde at 24 h post-ICH. The whole brain was collected and then fixed in 4% paraformaldehyde for another 24 h. Afterwards, the brain was fixed in 20% sucrose solution until the tissue sink to the bottom followed by 30% sucrose solution for another 24 h. After being frozen at − 25 °C, the brain was cut into 10-μm-thick coronal sections using a cryostat (CM1860; Leica Microsystems, Germany). To conduct double immunohistochemistry staining, the brain sections were incubated with primary antibody of anti-ionized calcium-binding adaptor molecule 1 (Iba-1, 1:100, Abcam, ab153696), anti-glial fibrillary acidic protein (GFAP, 1:200, CST, 3670, AB_561049), anti-von Willebrand factor (vWF, 1:50, Santa Cruz, sc-365712, AB_10842026), anti-NeuN (1:100, Abcam, ab104224, AB_10711040), and anti-Mc1r (1:50, Genetex, GTX108190) overnight at 4 °C. After being incubated with the appropriate secondary antibody (1:200, Bioss) at 37 °C for 1 h, the sections were visualized and photographed with a fluorescence microscope (U-HGLGPS, OLYMPUS, Japan). Microphotographs were analyzed with cellSens Standard software. The numbers of Iba-1-positive cells were identified and counted in three different fields in peri-hematoma area from five random coronal sections per brain, and data were expressed as cells/field.

### Western blotting

After mice were perfused with ice-cold PBS (0.1 M, pH 7.4) at 24 h post-operation, the peri-hematoma tissues were collected and stored in − 80 °C freezer until use. Western blotting was performed as previously described [28]. After sample preparation, equal amounts of protein were loaded onto an SDS-PAGE gel. After being electrophoresed and transferred to a PVDF membrane, the membrane was blocked 2 h at 37 °C followed by incubated with the primary antibody overnight at 4 °C. The primary antibodies were anti-Mc1r (1:1000, Abcam, ab180776), anti-Nr4a1 (1:500, Abcam, ab13851, AB_300679), anti-phospho-CREB (1:1000, cell signaling, 9198, Ser133, AB_2561044), anti-CREB (1:1000, cell signaling, 9197, AB_331277), anti-phospho-NF-κB p65 (1:1000, cell signaling, 3033, AB_331284), anti-NF-κB p65 (1:1000, cell signaling, 8242, AB_10859369), anti-IL-1β (1:1000, cell signaling, 31202), anti-TNF-α (1:1000, cell signaling, 11948, AB_2687962), anti-MMP-9 (1:500, Abcam, ab38898, AB_776512), anti-occludin (1:50000, abcam, ab167161, AB_2756463), anti-ZO-1 (1:1000, affinity, AF5145), anti-Lama5 (1:1000, abcam, ab184330), and anti-β-actin (1:5000, proteintech, 60008-1-Ig). The secondary antibodies (ZSGB-BIO) were incubated for 1 h at 37 °C. Immunoblots were then probed with an ECL Plus chemiluminescence reagent kit (4A Biotech) and visualized with the image system (Bio-Rad, Universal Hood III). All data were analyzed using the software ImageJ.

### Statistics analysis

All data were expressed as mean and standard deviation (mean ± SD). All analyses were performed using SigmaPlot 11.0 and GraphPad Prism 6 (GraphPad software, San Diego, CA, USA). Firstly, Shapiro-Wilk normality test was implemented in determining data normality. For the data that conformed to normal distribution, one-way ANOVA analysis followed by Tukey’s post hoc test was used for multiple-group comparisons. For the data that failed the normality test, Kruskal-Wallis one-way ANOVA on ranks, followed by Tukey’s multiple comparison post hoc analysis was performed. Statistical differences between two groups were analyzed using Student’s unpaired, two-tailed *t* test. *P* value of less than 0.05 was defined statistically significant (Additional file 2).

## Results

### Mortality and exclusion

The total mortality of ICH mice was 9.34% (17/182) in this study. None of the sham group mice died. There was no significant difference in mortality rate among the experimental groups. Six mice were ruled out from this study due to no hemorrhage (Additional file 1: Table S1).

### Expression of Mc1r after ICH

**Fig. 2:**
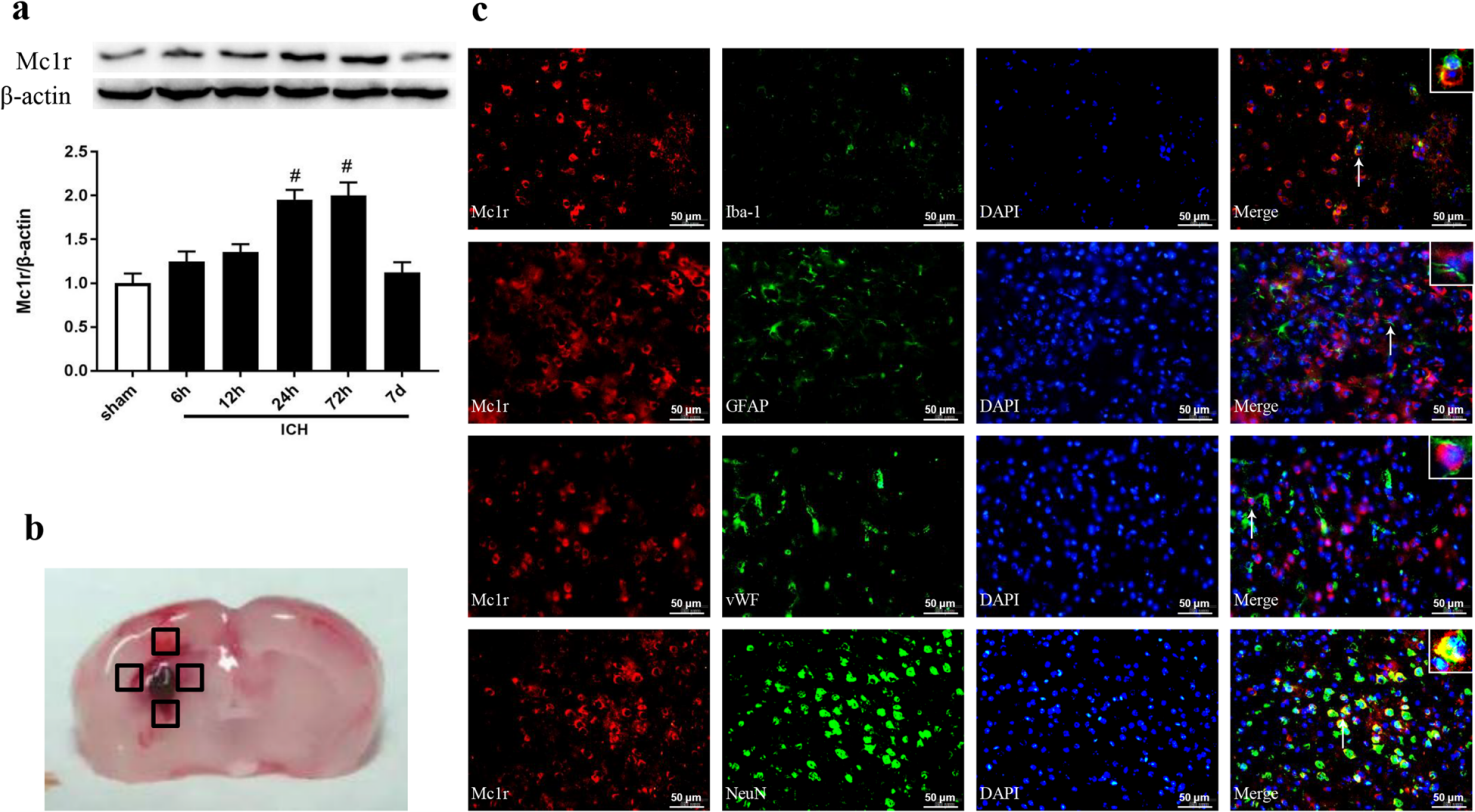
Expression of Mc1r after intracerebral hemorrhage (ICH). **a** Representative Western blot band and quantitative analyses of Mc1r time-dependent expression from the peri-hematoma tissue after ICH. ^#^*P* < 0.05 vs sham. *n* = 6 per group. **b** Representative brain sample with schematic illustration presenting the four regions in peri-hematoma area (indicated by black boxes). **c** Representative images of double immunofluorescence staining showed that Mc1r was colocalized with microglia (Iba-1), endothelium (vWF), astrocyte (GFAP), and neuron (NeuN) and at 24 h after ICH. *n* = 3 per group. Scale bar = 50 μm

As shown in Fig. 2a, the Mc1r expression in the peri-hematoma tissue was significantly increased at 24 h and reached its peak at 72 h after ICH, when compared to the sham group. Double immunofluorescence staining showed that Mc1r was mainly expressed in the microglia, astrocytes, and endothelial cells in the peri-hematoma tissue at 24 h after ICH (Fig. 2c).

### Administration of NDP-MSH improved neurological deficits and reduced brain edema and BBB permeability after ICH

**Fig. 3:**
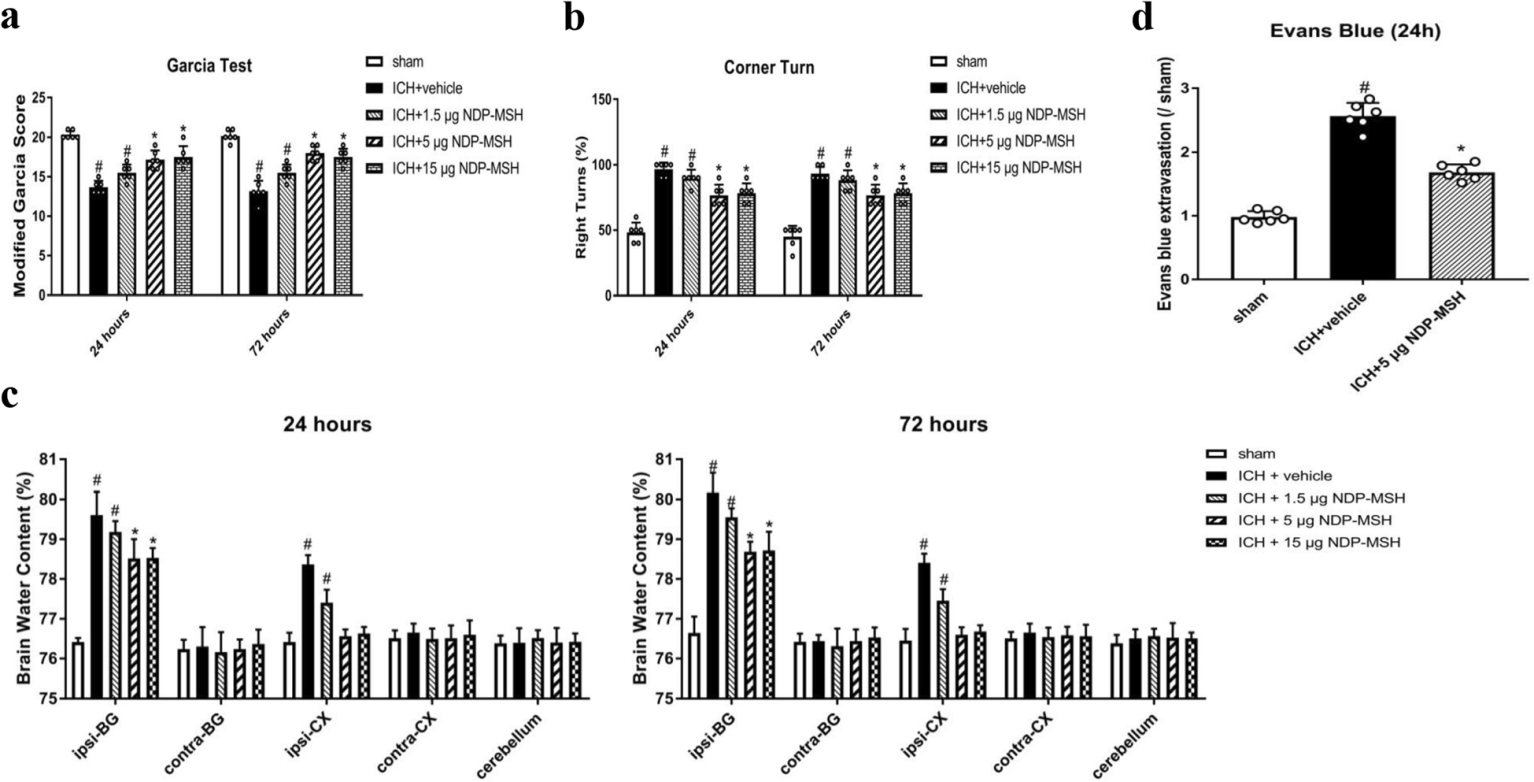
The neuroprotective effects of NDP-MSH on neurological functions, brain water content, and blood-brain barrier permeability after intracerebral hemorrhage (ICH). Treatment with NDP-MSH significantly improved neurological deficits (**a** and **b**) and reduced brain water content (**c**) at 24 and 72 h, as well as decreased EB extravasation at 24 h after ICH (**d**). *n* = 6 for each group. Brain sections were divided into five parts: ipsilateral basal ganglia (ipsi-BG), contralateral basal ganglia (contra-BG), ipsilateral cortex (ipsi-CX), contralateral cortex (contra-CX), and cerebellum. ^#^*P* < 0.05 vs sham; *P < 0.05 vs vehicle and NDP-MSH (1.5 μg)

The neurological deficits and brain edema were evidently worse at 24 and 72 h post-ICH in the ICH + vehicle and ICH + NDP-MSH (1.5 μg/mouse) groups, when compared with sham group. However, the administration of NDP-MSH (5 μg/mouse) and NDP-MSH (15 μg/mouse) significantly improved the neurological deficits (Fig. 3a, b) and reduced brain edema in ipsilateral basal ganglion (Fig. 3c). Based on these results, the optimal dose of NDP-MSH was 5 μg/mouse, which was used for the rest of the experiments. BBB permeability was assessed by EB extravasation in the right cerebral hemispheres. EB extravasation in the ICH + vehicle group was significantly increased at 24 h after ICH, whereas NDP-MSH treatment (5 μg/mouse) prominently decreased EB dye leakage compared with the ICH + vehicle group (Fig. 3d).

### Mc1r in vivo knockdown aggravated neurological deficits, brain edema, and BBB disruption after ICH

**Fig. 4:**
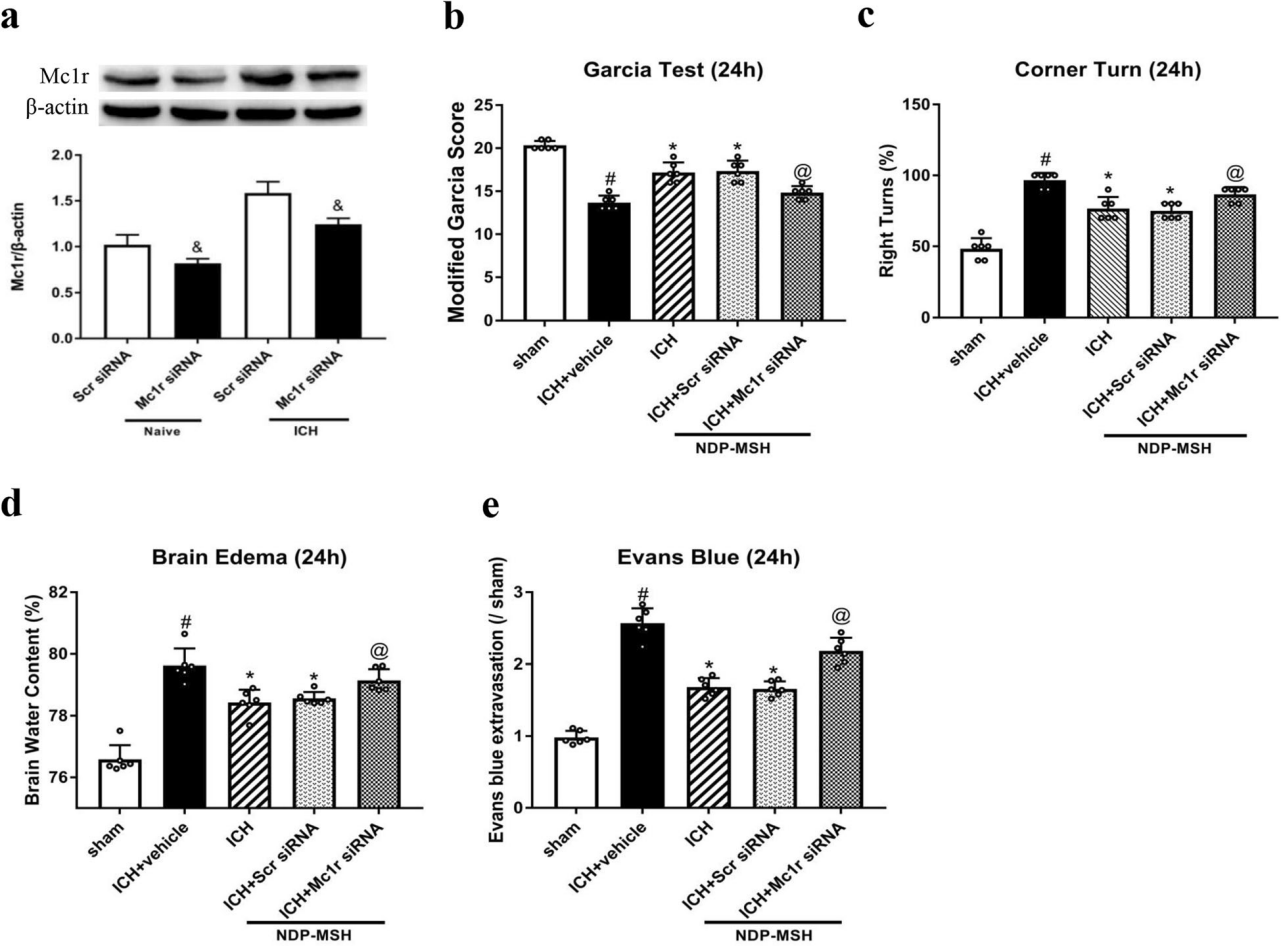
The effect of Mc1r siRNA on neurological functions, brain water content and BBB integrity at 24 h after ICH. **a** The expression of Mc1r was obviously reduced in the right hemisphere by Mc1r siRNA at 24 h post-ICH. ^&^*P* < 0.05 vs Scr siRNA. **b–e** Knockdown of Mc1r using Mc1r siRNA aggravated neurological deficits and increased brain edema and BBB permeability at 24 h following ICH. *n* = 6 per group. ^#^*P* < 0.05 vs sham; *P < 0.05 vs ICH + vehicle; ^@^*P* < 0.05 vs ICH + NDP-MSH, and ICH + NDP-MSH + Scr siRNA. Scr siRNA, scrambled siRNA

To further investigate, the protective role of NDP-MSH and Mc1r siRNA was administered by i.c.v injection to knockdown the expression of endogenous Mc1r. Western blot showed that the Mc1r expression was inhibited by Mc1r siRNA at 72 h after injection (Fig. 4a). The knockdown of Mc1r abolished the protective effect of NDP-MSH on neurological functions (Fig. 4b, c), brain edema (Fig. 4d), and BBB integrity (Fig. 4e) at 24 h post-ICH.

### Effect of NDP-MSH treatment and knockdown of Mc1r on the expression of downstream molecules after ICH

**Fig. 5:**
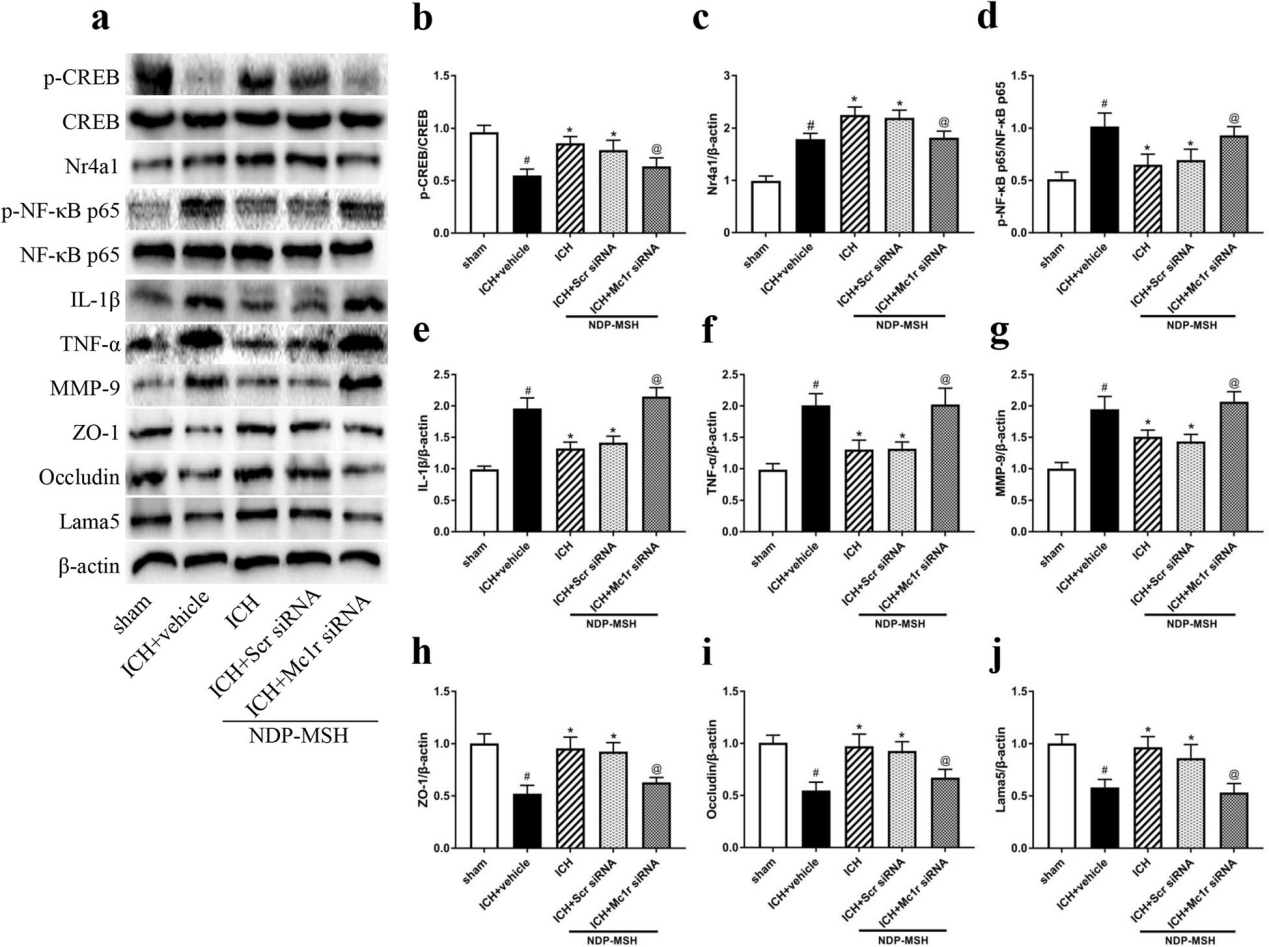
The effects of NDP-MSH treatment and knockdown of Mc1r on expression of downstream molecules at 24 h after ICH. **a** Representative Western blot bands of the downstream signaling pathway protein. **b**-**j** Densitometric quantification suggested that the administration of NDP-MSH, a agonist of Mc1r, prominently upregulated the levels of p-CREB, Nr4a1, ZO-1, occludin, and laminin-α5 (Lama5) at 24 h post-ICH . In addition, treatment with NDP-MSH significantly decreased p-NF-κB p65, IL-1β, TNF-α, and MMP-9 at the same time. In contrast, the knockdown of Mc1r led to a decrease of p-CREB, Nr4a1, ZO-1, occludin, and Lama5 and an increase of p-NF-κB p65, IL-1β, TNF-α, and MMP-9. ^#^*P* < 0.05 vs sham; *P < 0.05 vs ICH + vehicle; ^@^*P* < 0.05 vs ICH + NDP-MSH, and ICH + NDP-MSH + Scr siRNA. Scr siRNA, scrambled siRNA

Treatment with NDP-MSH increased phospho-CREB (p-CREB) expression in the peri-hematoma tissue at 24 h post-ICH, which increased the expression of downstream molecules including Nr4a1, ZO-1, occludin, and laminin-α5 (Lama5) and inhibited the expression of downstream inflammation-related proteins and MMP-9 (Fig. 5a-j), compared with ICH + vehicle group. In contrast, the knockdown of Mc1r using specific siRNA got opposite changes on the expression of downstream signaling molecules (Fig. 5a–j), compared with the ICH + NDP-MSH group.

### Treatment with NDP-MSH decreased microglial counts after ICH

**Fig. 6:**
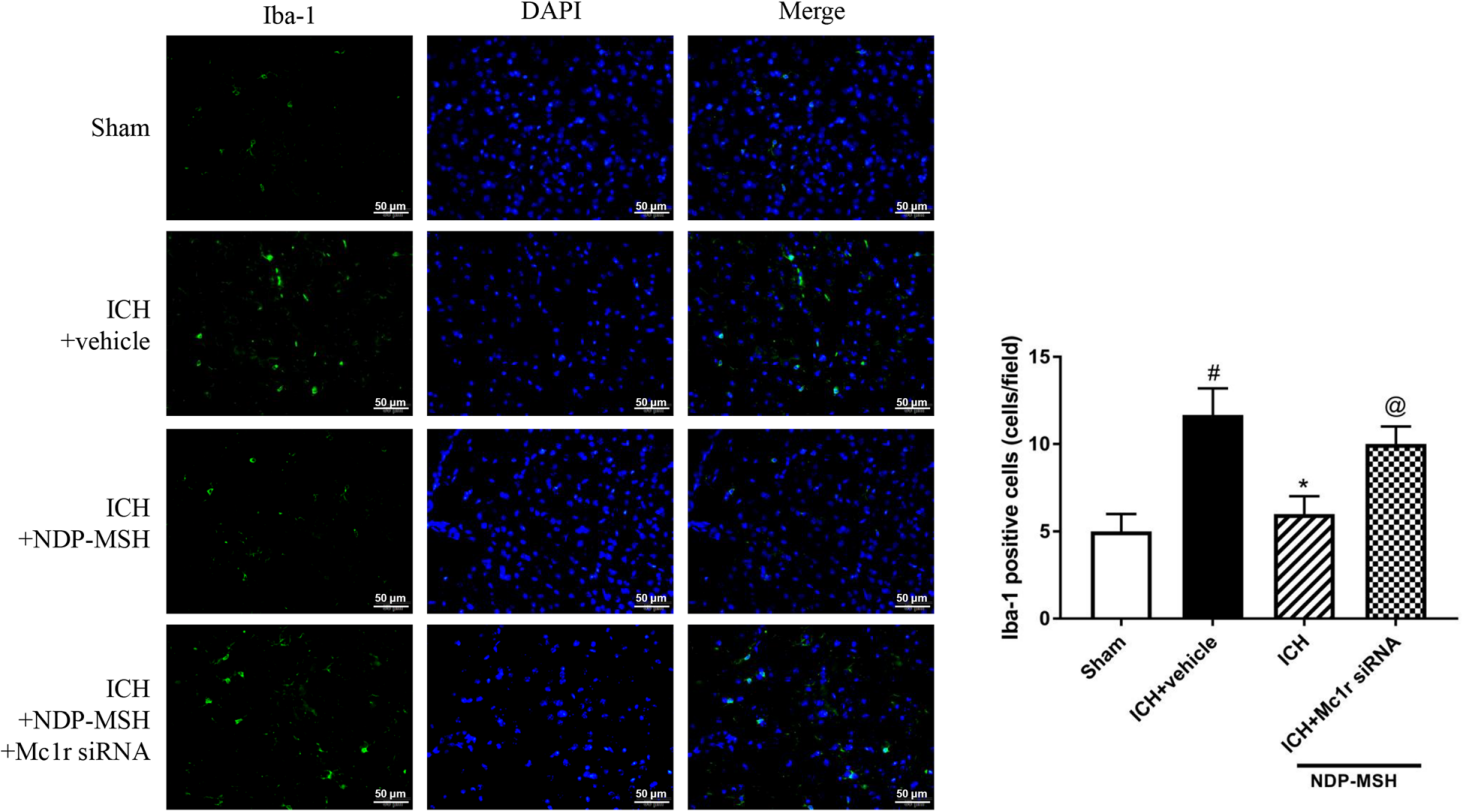
Microglial counts in the peri-hematoma area at 24 h following ICH. Representative microphotographs and quantification of Iba-1-stained microglia showed that NDP-MSH treatment reduced the number of Iba-1-positive cells, while this effect was reversed by Mc1r siRNA. ^#^*P* < 0.05 vs sham; *P < 0.05 vs ICH + vehicle; ^@^*P* < 0.05 vs ICH + NDP-MSH. *n* = 3 per group, scale bar = 50 μm

We investigated whether the anti-inflammatory function of NDP-MSH was associated with the decrease in the numbers of microglia in peri-hematoma tissue. As presented in Fig. 6, the numbers of Iba-1-positive cells were dramatically increased in ICH + vehicle group at 24 h post-ICH. The administration of NDP-MSH significantly reduced the number of Iba-1-positive cells, whereas the knockdown of Mc1r abolished this effect.

### Knockdown of Nr4a1 abolished the neuroprotective effects of NDP-MSH after ICH

**Fig. 7:**
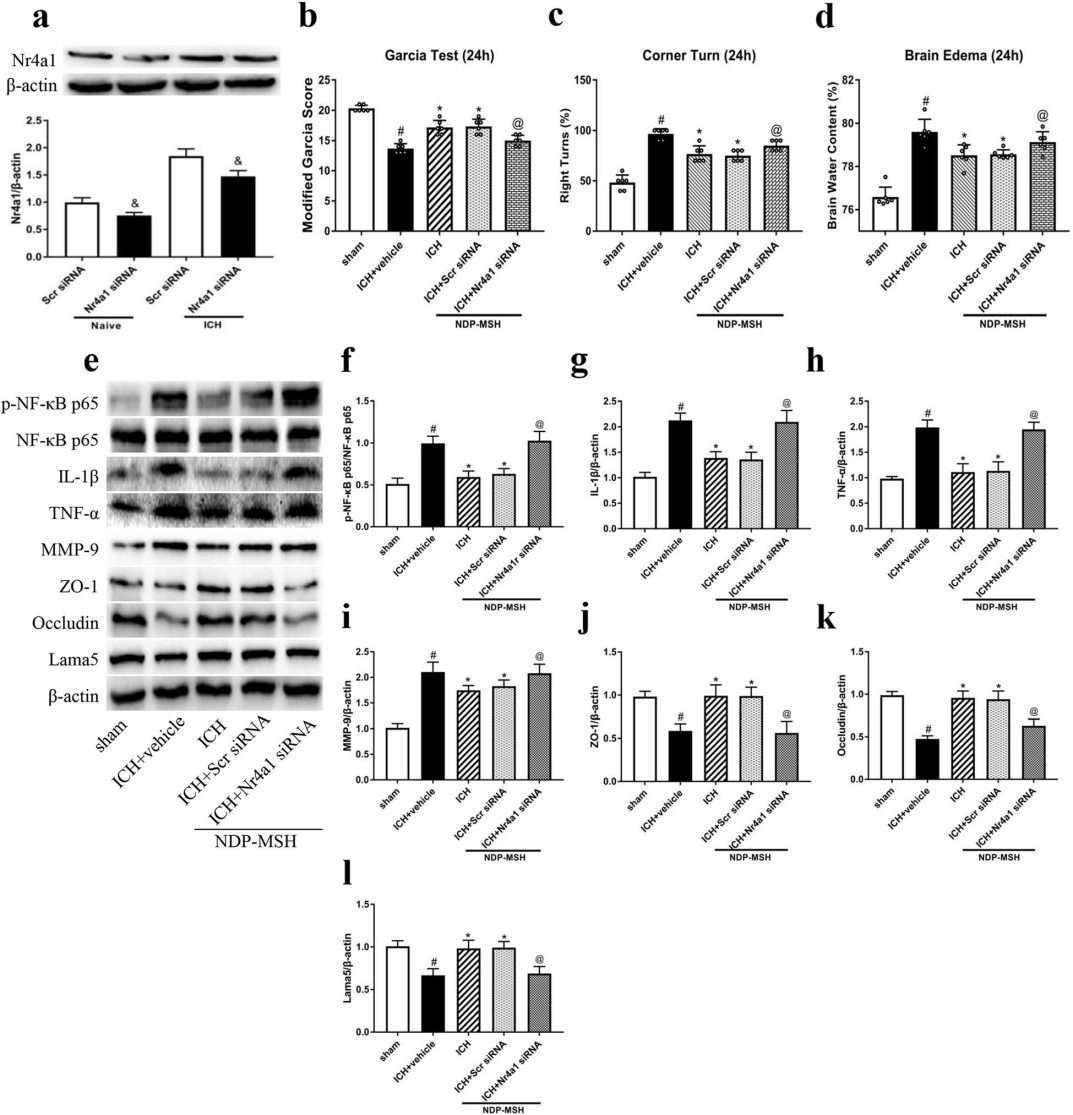
Knockdown of Nr4a1 reversed the neuroprotection of NDP-MSH following ICH. **a** The expression of Nr4a1 was significantly decreased in the right hemisphere by Nr4a1 siRNA at 24 h post-ICH. ^&^*P* < 0.05 vs Scr siRNA. **b–d** Knockdown of Nr4a1 aggravated neurological deficits and increased brain edema at 24 h following ICH. **e–l** Knockdown of Nr4a1 reversed the neuroprotection of NDP-MSH-induced change in protein levels of p-NF-κB p65, IL-1β, TNF-α, MMP-9, ZO-1, occludin, and Lama5 after ICH. ^#^*P* < 0.05 vs sham; *P < 0.05 vs ICH + vehicle; ^@^*P* < 0.05 vs ICH + NDP-MSH, and ICH + NDP-MSH + Scr siRNA. Scr siRNA, scrambled siRNA

To further determine whether the neuroprotective effects of NDP-MSH were regulated by Nr4a1, Nr4a1 siRNA was administered by i.c.v injection at 48 h before ICH induction and treated with NDP-MSH at 24 h post-ICH. Nr4a1 siRNA significantly decreased Nr4a1 expression at 72 h after injection (Fig. 7a). The knockdown of Nr4a1 exacerbated neurological impairments (Fig. 7b, c) and increased brain water content (Fig. 7d) at 24 h after ICH. Furthermore, the knockdown of Nr4a1 significantly increased the expression of p-NF-κB p65, IL-1β, TNF-α, and MMP-9 with a decrease of ZO-1, occludin, and Lama5 in the peri-hematoma tissue (Fig. 7e–l).

## Discussion

The novel findings in the present study were as follows: (1) Mc1r was significantly increased in the peri-hematoma tissue after ICH; (2) the administration of NDP-MSH attenuated brain edema and BBB disruption and improved neurological deficits following ICH; (3) treatment with NDP-MSH inhibited the expression of p-NF-κB p65, IL-1β, TNF-α, and MMP-9, as well as increased the expression of p-CREB, Nr4a1, ZO-1, occludin, and Lama5, thereby ameliorated brain injury post-ICH; (4) knockdown of Mc1r and Nr4a1 by specific siRNA aggravated neurological deficits, BBB damage, and inflammatory response after ICH; (5) CREB/Nr4a1/NF-κB signaling pathway was the potential mechanism of neuroprotection of NDP-MSH. Taken together, our findings indicated that NDP-MSH, by binding to Mc1r, attenuated neruoinflammation and BBB disruption after ICH, which is at least in part mediated by CREB/Nr4a1/NF-κB signaling pathway.

An ongoing body of researches demonstrated that inflammatory reaction and BBB disruption are critical factors to induce secondary brain injury following ICH [29, 30]. Following ICH, blood components rapidly enter the cerebral parenchyma and cause an inflammatory response. Furthermore, intensive inflammatory cascades aggravate BBB disruption, contribute to blood components infiltration into the brain in turn, and subsequently trap in a vicious circle to exacerbate brain injury after ICH.

Numerous studies have revealed that α-MSH analog NDP-MSH could inhibit inflammation and preserve BBB integrity [12, 13, 31]. In rat microglial cells, NDP-MSH exerted its anti-inflammatory effect by promoting a M2-like phenotype in microglia [31]. Following subarachnoid hemorrhage, treatment with NDP-MSH reduced vasospasm and inflammation through the decrease in the phosphorylation of extracellular-signal-regulated kinases (ERK1/2) [12]. Furthermore, NDP-MSH preserved BBB integrity and ameliorated neuroinflammation by preventing immune cell infiltration into the brain in mice with EAE through Mc1r/CREB/Nr4a1 signaling pathway [13]. Consistent with previous findings, our results revealed that treatment with NDP-MSH contributed to the upregulation of p-CREB, Nr4a1, ZO-1, occludin, Lama5, and downregulation of MMP-9 and inflammation-related molecules, and thus, attenuated neuroinflammation and BBB breakdown after ICH.

NDP-MSH exerts an anti-inflammatory effect by binding to different melanocortin receptors (Mc1r to Mc5r) [10, 13, 32, 33]. However, it has been proven that NDP-MSH has a specific higher affinity for Mc1r than other receptors [8, 10, 11]. Mc1r is widely distributed among various cell types, including macrophage, neutrophils, endothelial cells, and astrocytes [10]. In the present study, we observed that Mc1r was mainly expressed in the microglia, astrocytes, and endothelial cells after ICH. Moreover, the knockdown of Mc1r with Mc1r siRNA significantly abolished neuroprotective effects of NDP-MSH by increasing the expression of the inflammation-related molecules and MMP-9 and by decreasing the expression of ZO-1, occludin, and Lama5. Therefore, it is reasonable to speculate that Mc1r activation mediates NDP-MSH-induced neuroprotective effects after ICH. However, the finding was different from the previous observations, which showed that activating Mc4r with NDP-MSH or RO27-3225 could alleviate inflammatory reaction in the animal model of testicular ischemia and ICH [34, 35]. We supposed that such discrepancy may be due to the difference in animal models and tissue types.

Nr4a1 has been shown to inhibit inflammatory response by regulating the transcriptional activity of NF-κB [14, 18–20]. Nr4a1 also regulated microvessel permeability by increasing endothelial nitric-oxide synthase expression and by destabilizing endothelial junctions [36]. The NF-κB signaling pathway is well-known to be involved in mediating inflammatory response and BBB integrity after stroke [28, 37]. In the current study, the knockdown of Nr4a1 increased the expression of p-NF-κB p65, IL-1β, TNF-α, and MMP-9; decreased the expression of ZO-1, occludin, and Lama5; and resulted in neuroinflammation and BBB disruption. Therefore, knockdown of Nr4a1 reversed the neuroprotective roles of NDP-MSH.

There are some limitations in our study. NDP-MSH had been reported to possess multiple beneficial properties in a central nervous system disease, including anti-inflammation, anti-apoptosis, and anti-oxidation [13, 31, 34, 38, 39]. In this study, we only investigated the neuroprotective functions of NDP-MSH on neuroinflammation and BBB integrity after ICH. Thus, we cannot rule out the possibility that NDP-MSH-mediated anti-apoptosis and anti-oxidation may be involved in the neuroprotective effects after ICH. Further studies are needed to explore other functions of NDP-MSH after ICH and its underlying mechanisms. Second, we did not investigate the NDP-MSH-induced long-term neurological benefits following ICH. In addition, we only used male mice in this study. Thus, we cannot infer the effect of NDP-MSH on female mice after ICH.

## Conclusion

**Fig. 8:**
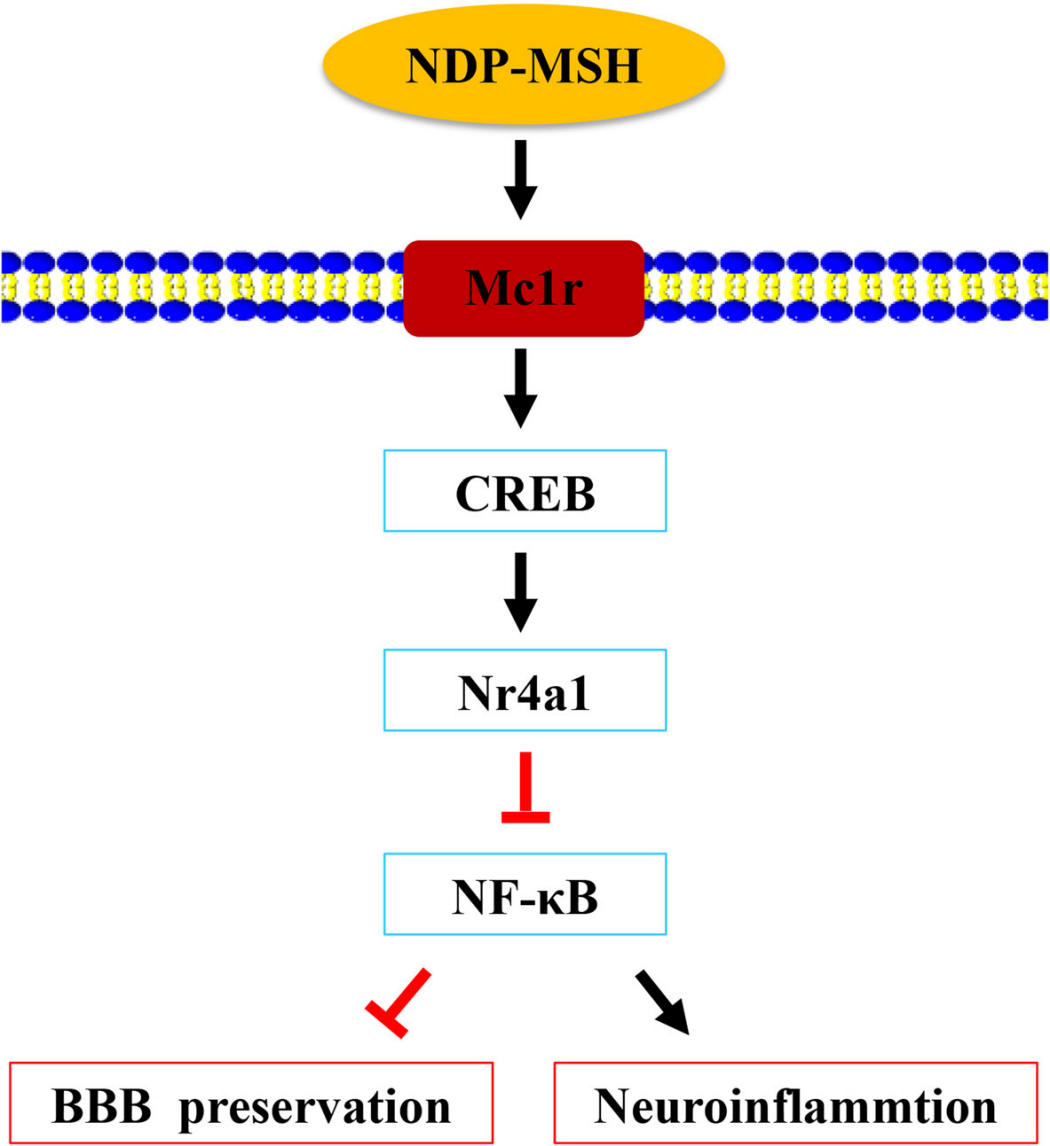
The schematic diagram of potential molecular mechanisms of neuroprotective effects of NDP-MSH through CREB/Nr4a1/NF-κB pathway after ICH

NDP-MSH binding Mc1r could alleviate neuroinflammation and BBB disruption and improve neurological impairments after ICH in mice. The neuroprotective role of NDP-SMH was mediated at least via CREB/Nr4a1/NF-κB signaling pathway (Fig. 8). Therefore, NDP-MSH might serve as a potential therapeutic agent against neuroinflammation for ICH patients.

## Supplementary information

**Additional file 1: Table S1.** Summary of experimental groups and mortality rate in the study.

**Additional file 2: Table S2.** The t statistic and degrees of freedom of results.

## Data Availability

The data used in the present study are available from the corresponding author on reasonable request.

## Abbreviations

BBB: Blood-brain barrier
CREB: cAMP response element-binding protein
GFAP: Glial fibrillary acidic protein
Iba-1: Ionized calcium-binding adaptor molecule 1
ICH: Intracerebral hemorrhage
Mc1r: Melanocortin-1 receptor
NDP-MSH: Nle^4^-D-Phe^7^-α-MSH
Nr4a1: Nuclear receptor subfamily 4 group A member 1
vWF: von Willebrand factor
α-MSH: α-Melanocyte-stimulating hormone
