# Supplementary material for: NDP-MSH binding melanocortin-1 receptor ameliorates neuroinflammation and BBB disruption through CREB/Nr4a1/NF-κB pathway after intracerebral hemorrhage in mice
Source: J Neuroinflammation. 2019 Oct 28;16:192. doi: 10.1186/s12974-019-1591-4 (PMC6816206; doi:10.1186/s12974-019-1591-4)
Supplement: Supplementary file 1 — Additional file 1: Table S1. Summary of experimental groups and mortality rate in the study. [file 12974_2019_1591_MOESM1_ESM.doc]

Table S1. Summary of experimental groups and mortality rate in the study

| **Experimental Groups** | **Neurological test**  **Brain water content** | **Evans blue** | **IF staining** | **WB** | **Exclusion** | **Mortality (%)** | **Subtotal** |
| --- | --- | --- | --- | --- | --- | --- | --- |
| **Experiment 1** |  |  |  |  |  |  |  |
| Sham |  |  |  | 6 | 0 | 0 | 6 |
| ICH (6 h, 12 h, 24 h, 72 h, 7d) |  |  | 3 | 6×5 | 2 | 5 (12.82%) | 40 |
| **Experiment 2** |  |  |  |  |  |  |  |
| sham | 6 | 6 |  |  |  |  | 12 |
| ICH+PBS | 6 | 6 |  |  | 1 | 2 (13.33%) | 15 |
| ICH+NDP-MSH (1.5μg) | 6 | 6 |  |  | 0 | 2 (14.29%) | 14 |
| ICH+NDP-MSH (5 μg) | 6 | 6 |  |  | 0 | 0 | 12 |
| ICH+NDP-MSH (15 μg) | 6 | 6 |  |  | 1 | 1 (7.14%) | 14 |
| **Experiment 3** |  |  |  |  |  |  |  |
| sham |  |  | 3 | 6 | 0 | 0 | 9 |
| ICH+PBS |  |  | 3 | 6 | 0 | 1 (10%) | 10 |
| ICH+NDP-MSH (5 μg) |  |  | 3 | 6 | 0 | 0 | 9 |
| ICH+NDP-MSH (5 μg)+Scr siRNA | 6 | 6 |  | 6 | 0 |  | 18 |
| ICH+Mc1r siRNA+NDP-MSH (5 μg) | 6 | 6 | 3 | 6 | 1 | 3 (12%) | 25 |
| Naive+Scr siRNA |  |  |  | 3 | 0 | 0 | 3 |
| Naive+Mc1r siRNA |  |  |  | 3 | 0 | 0 | 3 |
| ICH+Scr siRNA |  |  |  | 3 | 0 | 0 | 3 |
| ICH+Mc1r siRNA |  |  |  | 3 | 0 | 0 | 3 |
| **Experiment 4** |  |  |  |  |  |  |  |
| ICH+Nr4a1 siRNA+NDP-MSH (5 μg) | 6 |  |  | 6 | 1 | 2 (13.33%) | 15 |
| Naive+Nr4a1 siRNA |  |  |  | 3 | 0 | 0 | 3 |
| ICH+Nr4a1 siRNA |  |  |  | 3 | 0 | 1 (25%) | 4 |
| Total | 48 | 42 | 15 | 90 | 6 | 17 (9.34%) | 218 |

ICH, intracerebral hemorrhage. PBS, phosphate-buffered saline. Mc1r, melanocortin-1 receptor. Nr4a1, nuclear receptor subfamily 4 group A member 1. siRNA, small interfering RNA.
