# Supplementary material for: NDP-MSH binding melanocortin-1 receptor ameliorates neuroinflammation and BBB disruption through CREB/Nr4a1/NF-κB pathway after intracerebral hemorrhage in mice
Source: J Neuroinflammation. 2019 Oct 28;16:192. doi: 10.1186/s12974-019-1591-4 (PMC6816206; doi:10.1186/s12974-019-1591-4)
Supplement: Supplementary file 2 — Additional file 2: Table S2. The t statistic and degrees of freedom of results. [file 12974_2019_1591_MOESM2_ESM.doc]

Table S2 The t statistic and degrees of freedom of results

| Figures |  |  | Groups | t | df |
| --- | --- | --- | --- | --- | --- |
| Figure 2 |  |  | 24 h vs sham | 14.87 | 10 |
|  |  |  | 72 h vs sham | 13.04 | 10 |
| Figure 3 | **a** | 24 hours | ICH+vehicle vs sham | 13 | 5 |
|  |  |  | ICH+1.5μg NDP-MSH vs sham | 8.367 | 5 |
|  |  |  | ICH+5μg NDP-MSH vs ICH+vehicle | 8.174 | 5 |
|  |  |  | ICH+5μg NDP-MSH vs ICH+1.5μg NDP-MSH | 2.988 | 5 |
|  |  |  | ICH+15μg NDP-MSH vs ICH+vehicle | 5.861 | 5 |
|  |  | 72 hours | ICH+vehicle vs sham | 8.573 | 5 |
|  |  |  | ICH+1.5μg NDP-MSH vs sham | 9.439 | 5 |
|  |  |  | ICH+5μg NDP-MSH vs ICH+vehicle | 10.13 | 5 |
|  |  |  | ICH+5μg NDP-MSH vs ICH+1.5μg NDP-MSH | 3.727 | 5 |
|  |  |  | ICH+15μg NDP-MSH vs ICH+vehicle | 5.701 | 5 |
|  |  |  | ICH+15μg NDP-MSH vs ICH+1.5μg NDP-MSH | 4.472 | 5 |
|  | **b** | 24 hours | ICH+vehicle vs sham | 10.13 | 5 |
|  |  |  | ICH+1.5μg NDP-MSH vs sham | 8.73 | 5 |
|  |  |  | ICH+5μg NDP-MSH vs ICH+vehicle | 7.746 | 5 |
|  |  |  | ICH+5μg NDP-MSH vs ICH+1.5μg NDP-MSH | 3.162 | 5 |
|  |  |  | ICH+15μg NDP-MSH vs ICH+vehicle | 5.966 | 5 |
|  |  |  | ICH+15μg NDP-MSH vs ICH+1.5μg NDP-MSH | 2.907 | 5 |
|  |  | 72 hours | ICH+vehicle vs sham | 12.04 | 5 |
|  |  |  | ICH+1.5μg NDP-MSH vs sham | 13 | 5 |
|  |  |  | ICH+5μg NDP-MSH vs ICH+vehicle | 5 | 5 |
|  |  |  | ICH+5μg NDP-MSH vs ICH+1.5μg NDP-MSH | 2.907 | 5 |
|  |  |  | ICH+15μg NDP-MSH vs ICH+vehicle | 3.503 | 5 |
|  | **c** | ipsi-BG  24 hours | ICH+vehicle vs sham | 11.9 | 5 |
|  |  |  | ICH+1.5μg NDP-MSH vs sham | 20.21 | 5 |
|  |  |  | ICH+5μg NDP-MSH vs ICH+vehicle | 3.02 | 5 |
|  |  |  | ICH+5μg NDP-MSH vs ICH+1.5μg NDP-MSH | 2.812 | 5 |
|  |  |  | ICH+15μg NDP-MSH vs ICH+vehicle | 3.544 | 5 |
|  |  |  | ICH+15μg NDP-MSH vs ICH+1.5μg NDP-MSH | 3.509 | 5 |
|  |  | ipsi-CX  24 hours | ICH+vehicle vs sham | 13.39 | 5 |
|  |  |  | ICH+1.5μg NDP-MSH vs sham | 5.051 | 5 |
|  |  | ipsi-BG  72 hours | ICH+vehicle vs sham | 12.9 | 5 |
|  |  |  | ICH+1.5μg NDP-MSH vs sham | 19.67 | 5 |
|  |  |  | ICH+5μg NDP-MSH vs ICH+vehicle | 8.222 | 5 |
|  |  |  | ICH+5μg NDP-MSH vs ICH+1.5μg NDP-MSH | 7.831 | 5 |
|  |  |  | ICH+15μg NDP-MSH vs ICH+vehicle | 6.255 | 5 |
|  |  |  | ICH+15μg NDP-MSH vs ICH+1.5μg NDP-MSH | 3.522 | 5 |
|  |  | ipsi-CX  72 hours | ICH+vehicle vs sham | 12.56 | 5 |
|  |  |  | ICH+1.5μg NDP-MSH vs sham | 5.13 | 5 |
|  | **d** |  | ICH+vehicle vs sham | 17.15 | 10 |
|  |  |  | ICH+5μg NDP-MSH vs ICH+vehicle | 8.979 | 10 |
| Figure 4 | **a** | naive | Mc1r siRNA vs Scr siRNA | 2.901 | 4 |
|  |  | ICH | Mc1r siRNA vs Scr siRNA | 4.273 | 4 |
|  | **b** |  | ICH+vehicle vs sham | 16.9 | 10 |
|  |  |  | ICH+NDP-MSH vs ICH+vehicle | 6.069 | 10 |
|  |  |  | ICH+Scr siRNA+NDP-MSH vs ICH+vehicle | 6.149 | 10 |
|  |  |  | ICH+Mc1r siRNA+NDP-MSH vs ICH+NDP-MSH | 4.111 | 10 |
|  |  |  | ICH+Mc1r siRNA+NDP-MSH vs ICH+Scr siRNA+NDP-MSH | 4.294 | 10 |
|  | **c** |  | ICH+vehicle vs sham | 12.97 | 10 |
|  |  |  | ICH+NDP-MSH vs ICH+vehicle | 5.071 | 10 |
|  |  |  | ICH+Scr siRNA+NDP-MSH vs ICH+vehicle | 7.05 | 10 |
|  |  |  | ICH+Mc1r siRNA+NDP-MSH vs ICH+NDP-MSH | 2.535 | 10 |
|  |  |  | ICH+Mc1r siRNA+NDP-MSH vs ICH+Scr siRNA+NDP-MSH | 3.796 | 10 |
|  | **d** |  | ICH+vehicle vs sham | 10.47 | 10 |
|  |  |  | ICH+NDP-MSH vs ICH+vehicle | 4.295 | 10 |
|  |  |  | ICH+Mc1r siRNA+NDP-MSH vs ICH+NDP-MSH | 3.212 | 10 |
|  |  |  | ICH+Mc1r siRNA+NDP-MSH vs ICH+Scr siRNA+NDP-MSH | 3.468 | 10 |
|  | **e** |  | ICH+vehicle vs sham | 17.15 | 10 |
|  |  |  | ICH+NDP-MSH vs ICH+vehicle | 8.979 | 10 |
|  |  |  | ICH+Scr siRNA+NDP-MSH vs ICH+vehicle | 9.569 | 10 |
|  |  |  | ICH+Mc1r siRNA+NDP-MSH vs ICH+NDP-MSH | 5.557 | 10 |
|  |  |  | ICH+Mc1r siRNA+NDP-MSH vs ICH+Scr siRNA+NDP-MSH | 6.114 | 10 |
| Figure 5 | **b** |  | ICH+vehicle vs sham | 11.3 | 10 |
|  |  |  | ICH+NDP-MSH vs ICH+vehicle | 8.609 | 10 |
|  |  |  | ICH+Mc1r siRNA+NDP-MSH vs ICH+NDP-MSH | 5.234 | 10 |
|  |  |  | ICH+Mc1r siRNA+NDP-MSH vs ICH+Scr siRNA+NDP-MSH | 3.087 | 10 |
|  | **c** |  | ICH+vehicle vs sham | 13.81 | 10 |
|  |  |  | ICH+NDP-MSH vs ICH+vehicle | 6.017 | 10 |
|  |  |  | ICH+Mc1r siRNA+NDP-MSH vs ICH+NDP-MSH | 5.373 | 10 |
|  |  |  | ICH+Mc1r siRNA+NDP-MSH vs ICH+Scr siRNA+NDP-MSH | 4.768 | 10 |
|  | **d** |  | ICH+vehicle vs sham | 8.455 | 10 |
|  |  |  | ICH+NDP-MSH vs ICH+vehicle | 5.463 | 10 |
|  |  |  | ICH+Scr siRNA+NDP-MSH vs ICH+vehicle | 4.752 | 10 |
|  |  |  | ICH+Mc1r siRNA+NDP-MSH vs ICH+NDP-MSH | 5.216 | 10 |
|  |  |  | ICH+Mc1r siRNA+NDP-MSH vs ICH+Scr siRNA+NDP-MSH | 4.325 | 10 |
|  | **e** |  | ICH+vehicle vs sham | 13.54 | 10 |
|  |  |  | ICH+NDP-MSH vs ICH+vehicle | 7.932 | 10 |
|  |  |  | ICH+Scr siRNA+NDP-MSH vs ICH+vehicle | 6.757 | 10 |
|  |  |  | ICH+Mc1r siRNA+NDP-MSH vs ICH+NDP-MSH | 11.61 | 10 |
|  |  |  | ICH+Mc1r siRNA+NDP-MSH vs ICH+Scr siRNA+NDP-MSH | 10.27 | 10 |
|  | **f** |  | ICH+vehicle vs sham | 11.86 | 10 |
|  |  |  | ICH+NDP-MSH vs ICH+vehicle | 7.148 | 10 |
|  |  |  | ICH+Scr siRNA+NDP-MSH vs ICH+vehicle | 7.793 | 10 |
|  |  |  | ICH+Mc1r siRNA+NDP-MSH vs ICH+NDP-MSH | 5.84 | 10 |
|  |  |  | ICH+Mc1r siRNA+NDP-MSH vs ICH+Scr siRNA+NDP-MSH | 6.116 | 10 |
|  | **g** |  | ICH+vehicle vs sham | 10.24 | 10 |
|  |  |  | ICH+NDP-MSH vs ICH+vehicle | 4.657 | 10 |
|  |  |  | ICH+Scr siRNA+NDP-MSH vs ICH+vehicle | 5.377 | 10 |
|  |  |  | ICH+Mc1r siRNA+NDP-MSH vs ICH+NDP-MSH | 7.041 | 10 |
|  |  |  | ICH+Mc1r siRNA+NDP-MSH vs ICH+Scr siRNA+NDP-MSH | 7.845 | 10 |
|  | **h** |  | ICH+vehicle vs sham | 9.75 | 10 |
|  |  |  | ICH+NDP-MSH vs ICH+vehicle | 7.884 | 10 |
|  |  |  | ICH+Scr siRNA+NDP-MSH vs ICH+vehicle | 8.47 | 10 |
|  |  |  | ICH+Mc1r siRNA+NDP-MSH vs ICH+NDP-MSH | 6.721 | 10 |
|  |  |  | ICH+Mc1r siRNA+NDP-MSH vs ICH+Scr siRNA+NDP-MSH | 7.41 | 10 |
|  | **i** |  | ICH+vehicle vs sham | 10.4 | 10 |
|  |  |  | ICH+NDP-MSH vs ICH+vehicle | 7.405 | 10 |
|  |  |  | ICH+Scr siRNA+NDP-MSH vs ICH+vehicle | 7.824 | 10 |
|  |  |  | ICH+Mc1r siRNA+NDP-MSH vs ICH+NDP-MSH | 5.278 | 10 |
|  |  |  | ICH+Mc1r siRNA+NDP-MSH vs ICH+Scr siRNA+NDP-MSH | 5.315 | 10 |
|  | **j** |  | ICH+vehicle vs sham | 9.03 | 10 |
|  |  |  | ICH+NDP-MSH vs ICH+vehicle | 7.409 | 10 |
|  |  |  | ICH+Scr siRNA+NDP-MSH vs ICH+vehicle | 4.555 | 10 |
|  |  |  | ICH+Mc1r siRNA+NDP-MSH vs ICH+NDP-MSH | 7.962 | 10 |
|  |  |  | ICH+Mc1r siRNA+NDP-MSH vs ICH+Scr siRNA+NDP-MSH | 5.165 | 10 |
| Figure 6 |  |  | ICH+vehicle vs sham | 6.325 | 4 |
|  |  |  | ICH+NDP-MSH vs ICH+vehicle | 5.376 | 4 |
|  |  |  | ICH+Mc1r siRNA+NDP-MSH vs ICH+NDP-MSH | 4.899 | 4 |
| Figure 7 | **a** | naive | Nr4a1 siRNA vs Scr siRNA | 4.103 | 4 |
|  |  | ICH | Nr4a1 siRNA vs Scr siRNA | 3.819 | 4 |
|  | **b** |  | ICH+Nr4a1 siRNA+NDP-MSH vs ICH+NDP-MSH | 3.606 | 10 |
|  |  |  | ICH+Nr4a1 siRNA+NDP-MSH vs ICH+Scr siRNA+NDP-MSH | 3.796 | 10 |
|  | **c** |  | ICH+Nr4a1 siRNA+NDP-MSH vs ICH+Scr siRNA+NDP-MSH | 3.162 | 10 |
|  | **d** |  | ICH+Nr4a1 siRNA+NDP-MSH vs ICH+Scr siRNA+NDP-MSH | 2.646 | 10 |
|  | **f** |  | ICH+vehicle vs sham | 10.7 | 10 |
|  |  |  | ICH+NDP-MSH vs ICH+vehicle | 8.806 | 10 |
|  |  |  | ICH+Scr siRNA+NDP-MSH vs ICH+vehicle | 8.275 | 10 |
|  |  |  | ICH+Nr4a1 siRNA+NDP-MSH vs ICH+NDP-MSH | 8.168 | 10 |
|  |  |  | ICH+Nr4a1 siRNA+NDP-MSH vs ICH+Scr siRNA+NDP-MSH | 7.664 | 10 |
|  | **g** |  | ICH+vehicle vs sham | 16.12 | 10 |
|  |  |  | ICH+NDP-MSH vs ICH+vehicle | 9.499 | 10 |
|  |  |  | ICH+Scr siRNA+NDP-MSH vs ICH+vehicle | 9.292 | 10 |
|  |  |  | ICH+Nr4a1 siRNA+NDP-MSH vs ICH+NDP-MSH | 6.725 | 10 |
|  |  |  | ICH+Nr4a1 siRNA+NDP-MSH vs ICH+Scr siRNA+NDP-MSH | 6.772 | 10 |
|  | **h** |  | ICH+vehicle vs sham | 16.23 | 10 |
|  |  |  | ICH+NDP-MSH vs ICH+vehicle | 9.762 | 10 |
|  |  |  | ICH+Scr siRNA+NDP-MSH vs ICH+vehicle | 9.062 | 10 |
|  |  |  | ICH+Nr4a1 siRNA+NDP-MSH vs ICH+NDP-MSH | 9.418 | 10 |
|  |  |  | ICH+Nr4a1 siRNA+NDP-MSH vs ICH+Scr siRNA+NDP-MSH | 8.723 | 10 |
|  | **i** |  | ICH+vehicle vs sham | 12.67 | 10 |
|  |  |  | ICH+NDP-MSH vs ICH+vehicle | 4.078 | 10 |
|  |  |  | ICH+Scr siRNA+NDP-MSH vs ICH+vehicle | 2.977 | 10 |
|  |  |  | ICH+Nr4a1 siRNA+NDP-MSH vs ICH+NDP-MSH | 4.071 | 10 |
|  |  |  | ICH+Nr4a1 siRNA+NDP-MSH vs ICH+Scr siRNA+NDP-MSH | 2.884 | 10 |
|  | **j** |  | ICH+vehicle vs sham | 9.396 | 10 |
|  |  |  | ICH+NDP-MSH vs ICH+vehicle | 6.581 | 10 |
|  |  |  | ICH+Scr siRNA+NDP-MSH vs ICH+vehicle | 7.571 | 10 |
|  |  |  | ICH+Nr4a1 siRNA+NDP-MSH vs ICH+NDP-MSH | 5.693 | 10 |
|  |  |  | ICH+Nr4a1 siRNA+NDP-MSH vs ICH+Scr siRNA+NDP-MSH | 6.216 | 10 |
|  | **k** |  | ICH+vehicle vs sham | 21.8 | 10 |
|  |  |  | ICH+NDP-MSH vs ICH+vehicle | 13.18 | 10 |
|  |  |  | ICH+Scr siRNA+NDP-MSH vs ICH+vehicle | 10.83 | 10 |
|  |  |  | ICH+Nr4a1 siRNA+NDP-MSH vs ICH+NDP-MSH | 7.087 | 10 |
|  |  |  | ICH+Nr4a1 siRNA+NDP-MSH vs ICH+Scr siRNA+NDP-MSH | 6.041 | 10 |
|  | **l** |  | ICH+vehicle vs sham | 8.221 | 10 |
|  |  |  | ICH+NDP-MSH vs ICH+vehicle | 6.24 | 10 |
|  |  |  | ICH+Scr siRNA+NDP-MSH vs ICH+vehicle | 7.442 | 10 |
|  |  |  | ICH+Nr4a1 siRNA+NDP-MSH vs ICH+NDP-MSH | 5.698 | 10 |
|  |  |  | ICH+Nr4a1 siRNA+NDP-MSH vs ICH+Scr siRNA+NDP-MSH | 6.762 | 10 |
